# Expression Analysis of the *Theileria parva* Subtelomere-Encoded Variable Secreted Protein Gene Family

**DOI:** 10.1371/journal.pone.0004839

**Published:** 2009-03-27

**Authors:** Jacqueline Schmuckli-Maurer, Carlo Casanova, Stéfanie Schmied, Sarah Affentranger, Iana Parvanova, Simon Kang'a, Vishvanath Nene, Frank Katzer, Declan McKeever, Joachim Müller, Richard Bishop, Arnab Pain, Dirk A. E. Dobbelaere

**Affiliations:** 1 Molecular Pathobiology, Vetsuisse Faculty, University of Bern, Bern, Switzerland; 2 The Institute for Genomic Research (TIGR), Rockville, Maryland, United States of America; 3 Centre for Tropical Veterinary Medicine, Royal (Dick) School of Veterinary Studies, University of Edinburgh, Easter Bush Veterinary Centre, Roslin, Midlothian, United Kingdom; 4 Institute of Parasitology, University of Bern, Bern, Switzerland; 5 International Livestock Research Institute, Nairobi, Kenya; 6 Sanger Institute, Wellcome Trust Genome Campus, Hinxton, Cambridge, United Kingdom; Federal University of São Paulo, Brazil

## Abstract

**Background:**

The intracellular protozoan parasite *Theileria parva* transforms bovine lymphocytes inducing uncontrolled proliferation. Proteins released from the parasite are assumed to contribute to phenotypic changes of the host cell and parasite persistence. With 85 members, genes encoding *s*ubtelomeric *v*ariable *s*ecreted *p*roteins (*SVSP*s) form the largest gene family in *T. parva*. The majority of *SVSP*s contain predicted signal peptides, suggesting secretion into the host cell cytoplasm.

**Methodology/Principal Findings:**

We analysed *SVSP* expression in *T. parva*-transformed cell lines established *in vitro* by infection of T or B lymphocytes with cloned *T. parva* parasites. Microarray and quantitative real-time PCR analysis revealed mRNA expression for a wide range of *SVSP* genes. The pattern of mRNA expression was largely defined by the parasite genotype and not by host background or cell type, and found to be relatively stable *in vitro* over a period of two months. Interestingly, immunofluorescence analysis carried out on cell lines established from a cloned parasite showed that expression of a single SVSP encoded by *TP03_0882* is limited to only a small percentage of parasites. Epitope-tagged *TP03_0882* expressed in mammalian cells was found to translocate into the nucleus, a process that could be attributed to two different nuclear localisation signals.

**Conclusions:**

Our analysis reveals a complex pattern of *Theileria SVSP* mRNA expression, which depends on the parasite genotype. Whereas in cell lines established from a cloned parasite transcripts can be found corresponding to a wide range of *SVSP* genes, only a minority of parasites appear to express a particular *SVSP* protein. The fact that a number of SVSPs contain functional nuclear localisation signals suggests that proteins released from the parasite could contribute to phenotypic changes of the host cell. This initial characterisation will facilitate future studies on the regulation of *SVSP* gene expression and the potential biological role of these enigmatic proteins.

## Introduction

The subtelomeres of many pathogenic microorganisms contain gene families involved in host-pathogen interactions such as adherence, invasion or escape from immunity (reviewed in [1]). Well-documented examples include the *var* genes in *Plasmodium falciparum* [2], the *vsg* genes in *Trypanosoma brucei* [3] and the *EPA* genes in the pathogenic fungus *Candida glabrata* [4]. The location in telomere-associated regions allows special mechanisms to regulate gene expression. In addition telomere clustering at the nuclear periphery can promote ectopic recombination in telomere-proximal gene families leading to antigenic variation [1]. Heterochromatin, the condensed and transcriptionally inactive form of chromatin, is constitutively present at telomeres and the resulting transcriptional silencing has been described as “telomere position effect” [5]. Post-translational modifications of histones can regulate the extent of condensation and heterochromatin-mediated regulation is one mechanism used by many pathogens to control differential expression of members of subtelomeric gene families [6]. In *P. falciparum*, for instance, the exclusive and alternating expression of one single *var* gene at a time is under heterochromatin-mediated control [2].

The protozoan parasites *Theileria parva* and *Theileria annulata*, like *Plasmodium* spp., belong to the phylum *Apicomplexa*. Both *Theileria* parasites are transmitted by *ixodid* ticks, causing fatal cattle diseases in large parts of Africa and Asia, respectively. After invasion of bovine lymphocytes, *Theileria* sporozoites rapidly eliminate the enclosing host cell membrane and over the next two to three days, the parasite-now free in the cytoplasm-differentiates into a multinucleated schizont [7]. *T. parva* and *T. annulata* schizonts have the unique ability among eukaryotic microorganisms to convert the host cells they infect into a transformed state. This is accompanied by uncontrolled proliferation and resistance to apoptosis. *Theileria*-transformed cells also acquire the capacity to metastasise (reviewed in [8], [9]).

While the host signalling pathways mediating these phenotypic alterations have been studied in considerable detail, the parasite factors involved are largely unknown (reviewed in [10], [11], [12], [13]. In the cytoplasm, the parasite is perfectly positioned to interfere with host cell pathways that regulate proliferation and survival. This can involve events that occur at the parasite surface, as shown for the NF-κB activation pathway [14], or potentially also through secreted parasite proteins [15], [16], [17].

Secreted proteins are of particular interest as they theoretically have the highest potential for processing by the proteasome and presentation of peptides in association with bovine host MHC Class I molecules. The elimination of *T. parva*-infected lymphocytes by MHC class I-restricted cytotoxic T cell responses is a proven effector mechanism for protective immunity in cattle [18], [19]. Therefore such proteins could also function as vaccine antigens and there is preliminary evidence to support this [20], [21].

While screening a *T. parva* library using the yeast-2-hybrid system, we made the chance discovery of several members of a novel gene family encoding glutamine (Q)- and proline (P)-rich proteins, most of which contained putative signal peptides for secretion. These proteins were distinct from PIM (polymorphic immunodominant molecule), also called QP-protein [22], [23]. Upon completion of the *T. parva* and *T. annulata* genome sequences [24], [25] it became clear that the identified genes were part of a large and unique family located in a telomere-associated region of all four *Theileria* chromosomes. The gene family was originally designated subtelomeric variable secreted protein (*SVSP*) gene family. We suggest to designate the family ‘subtelomere-*encoded* variable secreted proteins’ (*SVSP*) because the genes (rather than the proteins) are clearly subtelomeric and there is no evidence that the (SVSP) proteins themselves localise to subtelomeric regions. With 85 members in *T. parva* and 48 members in *T. annulata*, it is the largest family observed in these organisms. Between 2 and 26 genes within this family are located at each of the eight chromosome ends, arranged in tandem arrays with very short intergenic regions of about 200 to 300 base pairs and aligned in centromeric-telomeric direction [24], [25]. The organisation of the *SVSP* genes is schematically presented in Fig. S1. An analysis using SignalP software [26] predicts that many *SVSP*s have a signal peptide for access to the parasite secretory pathway (Table S2) [25].

**Figure 1:**
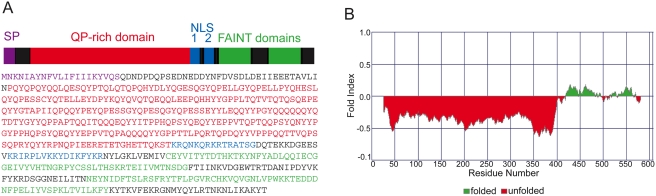
Schematic presentation of *TP08_0882*, a typical *SVSP.* A. Schematic view and amino acid sequence of *TP03_0882*. *TP03_0882* is 607 amino acids long. The polypeptide has a putative signal peptide (SP) for secretion (purple) with a cleavage site after residue 21 (predicted by the SignalP3.0 web server). A large N-terminal region containing abundant Q and P residues (red) is followed by C-terminal region containing two nuclear localisation signals (NLS 1 and 2, blue) and two FAINT domains from aa 422 to 481 and aa 520 to 579 (green). B. Analysis of the *TP03_0882* protein using the FoldIndex© software. The N-terminal region of the protein is intrinsically unfolded, while the conserve C-terminal region is predicted to fold.

The amino acid sequence of a *SVSP* (*TP03_0882*) containing the characteristic features of this family is presented in Figure 1A. The general structure of *SVSP*s consists of a short conserved N-terminal region, in most cases containing a putative signal peptide for secretion, followed by a QP-rich region which is predicted to be highly unstructured (Fig. 1B) [27]. The conserved C-terminus has no significant identity to known proteins and nearly all *SVSP* molecules contain *Theileria*-specific, highly divergent domains termed ‘frequently associated in *Theileria*’ (FAINT). The function of these domains that contain approximately 70 amino acid residues is currently unknown [25].

Information obtained from analysis of the *T. parva* transcriptome by massively parallel signature sequencing (MPSS) technology combined with the fact that we isolated several *SVSP* clones from a cDNA library indicates that many *SVSP* genes are transcribed in the schizont stage ([25], [28] and data not shown).

The analysis by MPSS of *SVSP* transcripts in a cell line transformed by *T. parva* (Muguga) [29] suggested a mosaic-like expression pattern (see Fig. S1 for an overview of *SVSP* gene organisation and expression as reported by Bishop *et al*. [28]). Allocating transcripts from a single infected cell line to individual genes, those expressed at low levels (4-99 transcripts per million) were found to alternate with genes expressed at medium levels (100-999 transcripts per million), and transcribed genes were interspersed with *SVSP* genes for which no transcripts were detectable.

The present paper provides an initial characterisation of this unusual *T. parva* gene family. We analysed the expression of *SVSP* genes in a set of phenotypically different cell lines, established by infection with cloned *T. parva* parasites, to investigate whether the pattern of expression is conserved or changes depending on parasite genotype, or host cell type and background. Using anti-*SVSP* antibodies, we present the first evidence for *SVSP* protein expression. By transfecting mammalian cells with different *SVSP* constructs, we show that the nuclear localisation signals (NLS) found in several *SVSP*s are functional and can target *SVSP*s to different nuclear compartments.

## Results

### Expression patterns of *SVSP* genes in different *T. parva*-infected cloned cell lines

In order to investigate whether *SVSP* genes are differentially expressed depending on the host cell type the parasite resides in, we compared transcript levels in a CD4+ T cell line (211T-A3) and a B cell line (211B-A3) originating from the animal 211 that was infected with the *T. parva* (Marikebuni) clone A3 [30]. A microarray was used that contained 70-mer probes corresponding to 78 of the 85 *SVSP* genes. Seven *SVSP* genes were not represented on the microarray because appropriate probes could not be designed due to the presence of low complexity regions, lack of uniqueness and/or unsuitable GC content falling outside the required range (Kang'a *et al.*, in preparation). Of the 78 *SVSP*s analyzed, transcripts of 56 genes were detected (Table S1 and Fig. S1). At the different subtelomeres, the 22 genes with no detectable transcripts were interspersed between transcribed *SVSP*s. For a block of 9 genes on the reverse strand of chromosome 3, evidence for transcription was absent. However, this region spanning loci *TP03_0871* to *TP03_0880* also includes 4 genes that were not represented on the microarrays. Two of these 4 genes share 100% identity with another gene (TP03_0871 is identical to TP03_0884; TP03_0879 is identical to TP03_0874) and TP03_0873 is identical to TP03_0878 except for the first 27 bp. As no transcripts were detected for TP03_0874 and TP03_0878 and as probes were designed corresponding to 3′ regions of the genes it can be concluded that also TP03_0879 and TP03_0873 are not expressed. Because TP03_0871 is identical to TP03_0884, the transcript detected by microarray analysis suggests that one or both genes are expressed. *SVSP* genes for which no transcripts could be detected were located at all subtelomeres except the reverse strands of chromosomes 1 and 4. Genes with log2 ratios higher than 0.9 (which corresponds to an approximately two-fold difference) were considered as differentially expressed. When the T- and B-cell lines (211T-A3 and 211B-A3) transformed by *T. parva* (Marikebuni) A3 were compared, for most of the genes no significant difference in transcript level could be detected. Only two genes, *TP02_0954* and *TP03_0890*, showed slightly elevated transcript levels in the T cell line (log2 ratios of 0.9 and 1.4, respectively; Table S1).

Next, *SVSP* expression in the two *T. parva* (Marikebuni) A3-infected cell lines was compared to that for a third cell line, 951T-F44, consisting of CD4+ T cells (derived from animal 951) transformed by the *T. parva* (Marikebuni) clone F44 30, 31. Compared to the cells transformed with the A3 clone, transcript levels for the majority of *SVSP* genes were largely conserved in the cell line 951T-F44. However, there were eight genes for which differential expression above the threshold of a log2 ratio of 0.9 was detected (Table S1, Fig. S1). These initial findings indicated that multiple *SVSP* genes are transcribed simultaneously in cell lines obtained by transformation of T and B cells with cloned *T. parva* (Marikebuni) parasites. A small percentage of *SVSP* genes, however, also appear to be differentially expressed.

**Figure 2:**
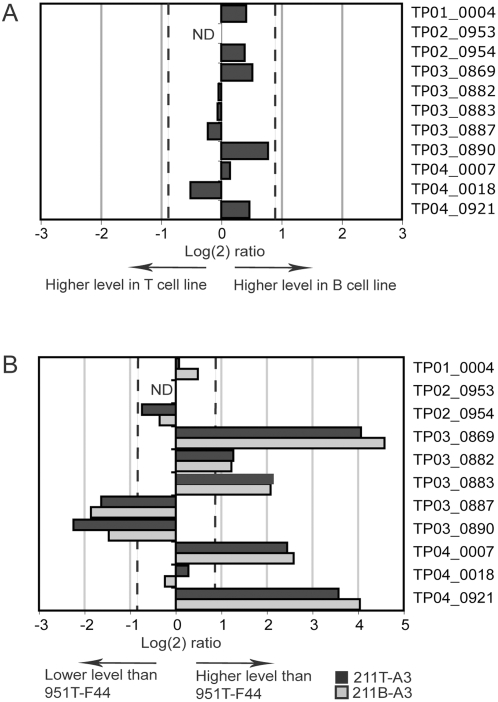
Quantitative RT-PCR analysis of *SVSP* expression patterns in different *T. parva*-infected cell lines. A. Comparison of *SVSP* transcript levels in T and B cell lines transformed with the *T. parva* (Marikebuni) A3 clone. Cells originated from the same animal (211). Relative transcript levels for 11 selected *SVSP* genes (listed on the right) were assessed by qRT-PCR in the cell lines 211T-A3 and 211B-A3. Dashed lines indicate log(2) ratios of 0.9 and -0.9, the arbitrarily defined Index threshold used for microarray analysis above which differential expression of genes is considered significant. For one gene no transcripts were detected (ND). B. *SVSP* transcript levels in the *T. parva* (Marikebuni) A3-infected cell lines 211T-A3 (dark bars) and 211B-A3 (pale bars) compared to a third cell line 951T-F44, infected with a different *T. parva* (Marikebuni) clone (F44).

**Figure 3:**
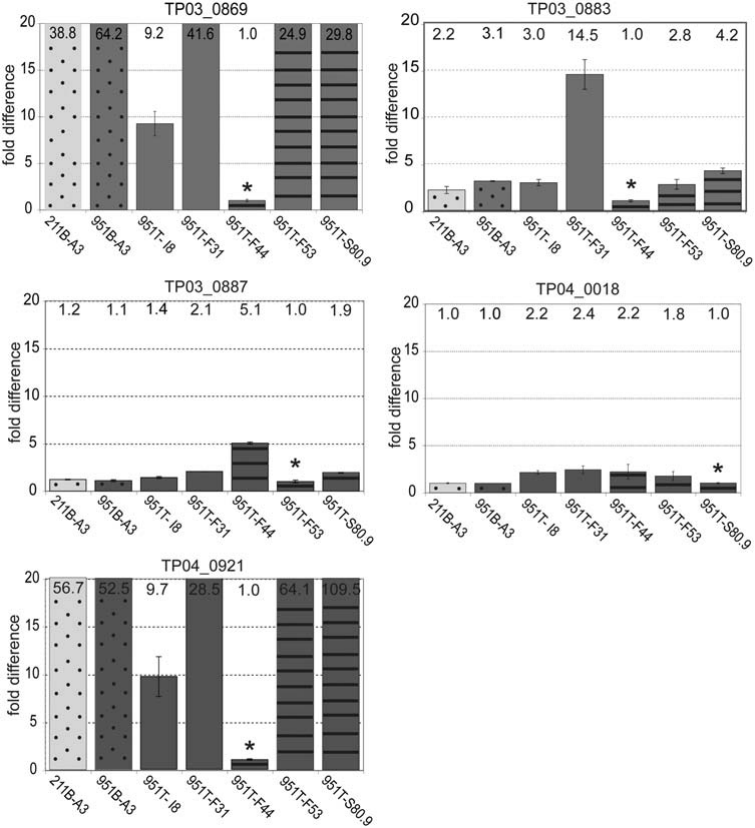
qRT-PCR analysis of five *SVSP* genes in seven different cloned *T. parva*–infected cell lines. The expression of five *SVSP* genes, *TP03_0869*, *TP03_0883*, *TP03_0887*, *TP04_0018* and *TP04_0921* was monitored in seven cell lines transformed by different *T. parva* clones (see Table 2 for cell lines). For each gene and each cell line, the relative fold difference in relation to the cell line with the lowest transcript level of the respective gene (indicated as 1.0, labeled with *) is indicated. Data represent the mean of the transcript levels from two independent culture flasks. The actual fold difference is indicated at the top of the bar. Error bars denote the standard error of the mean. Differently coloured bars indicate cell lines from different animals. Dotted bars indicate B cell lines, bars with no pattern indicate γδ T cells, and CD4+ T cells are indicated with striped bars.

In order to consolidate the observations obtained using microarrays, we performed quantitative real-time PCR (qRT-PCR) and monitored the expression of 11 *SVSP* genes using the same total RNA that had been prepared for the microarray analysis. In addition to the genes *TP02_0954* and *TP03_0890* we analyzed two genes for which no transcripts were detected in microarray experiments (*TP02_0953* and *TP04_0018*), one gene for which no differential expression was observed in all three cell lines (*TP03_0882*) and the genes *TP01_0004*, *TP03_0869*, *TP03_0883*, *TP03_0887*, *TP04_0007* and *TP04_0921*, which, according to microarray analysis, were differentially expressed in the cell line 951T-F44. Transcript levels of these eleven genes were compared in the cell lines 211T-A3 and 211B-A3. For the eleven *SVSP*s analyzed, no significant difference in transcript levels could be observed when CD4+ T cells or B cells transformed by the *T. parva* (Marikebuni) A3 clone were compared (Fig. 2A). A schematic overview of the position of these genes at the telomeres of chromosomes 1 to 4 is shown in Fig. S1. The small differences in transcript levels of the genes *TP02_0954* and *TP03_0890* observed by microarray analysis could not be confirmed. In relation to the cell line 951T-F44, the overall *SVSP* expression pattern in 211T-A3 and 211B-A3 was highly similar (Fig. 2B). For single *SVSP* genes, however, transcript levels observed in the cell line 951T-F44 differed clearly from that observed for the other two cell lines (Fig. 2B), with some genes showing higher and other genes lower quantities of steady state mRNA. As the cell line 951T-F44 is derived from a different animal and harbours a different parasite clone, it was not clear whether the altered expression pattern could be attributed to the host background, or parasite genotype. To address this question, relative expression levels of five selected *SVSP* genes were assessed in seven different *T. parva*-infected cell lines. These included *T. parva* (Marikebuni) A3 in B cells of two different animals (211B-A3 and 951B-A3), two different *T. parva* (Marikebuni) clones in γδ T cells of the same animal (951T-I8 and 951T-F31), and three CD4+ T cell lines, each harbouring one of two different *T. parva* (Marikebuni) clones or a *T. parva* (Muguga) clone (951T-F44, 951T-F53 and 951T-S80.9) (Table 2). For the five genes analyzed, no difference in transcript levels (with more than two-fold difference) was observed when *T. parva* (Marikebuni) A3 parasitizing B cells from two different animals was compared (Fig. 3, first two bars in all five panels). By contrast, pronounced differences were detected in cell lines infected with different parasite clones. For instance, in infected CD4+ T cell lines of animal 951, transcript levels of *TP03_0869* and *TP04_0921* are much lower in the clone *T. parva* (Marikebuni) F44 than in *T. parva* (Marikebuni) F53 (to a maximum of an approximately 64-fold difference), while transcript levels of these two genes are comparable in the *T. parva* (Marikebuni) clone F53 and the *T. parva* (Muguga) clone S80.9 (Fig. 3, last two bars). On the other hand, *TP03_0887* transcript levels are five times higher in *T. parva* (Marikebuni) F44 than in *T. parva* (Marikebuni) F53. *T. parva* (Marikebuni) F31 in the γδ T cell line of animal 951 has an approximately five-fold higher level of *TP03_0883* transcripts than *T. parva* (Marikebuni) I8 on the same host cell background (Fig. 3).

As *TP03_0877* belongs to a small region for which specific probes for microarray analysis were not available, transcript levels were also analysed by qRT-PCR in the cell lines 951B-A3 and 951T-F44. With a log2 ratio of 1, the mRNA expression level was found to be slightly elevated in 951T-F44 compared to 951B-A3 (Fig. S1). This data is not included in Fig. 2B as different RNA preparations were used than for the microarray analysis.

Sub-telomeric regions are often highly polymorphic [32] and this has been demonstrated for *T. parva* ORFs located in telomere-associated DNA [33]. In order to exclude the possibility that the observed differences in transcript levels are caused by suboptimal hybridisation of the microarray probes or the PCR primers due to polymorphisms in different parasite genotypes, we sequenced selected gene fragments from different cell lines. Fragments of approximately 1000 bp encompassing the target sites for microarray probes and qRT-PCR primers in the genes *TP03_0869*, *TP03_0883* and *TP04_0921* of the cell lines 951-A3, 951T-F44, 951T-F31, 951-I8 and 951T-S80.9 were sequenced. Apart from one single nucleotide not affecting the hybridization region, sequences were identical in all cell lines (data not shown).

To test whether the pattern of *SVSP* expression is stable, transcript levels of *TP03_0869, TP04_0018* and *TP04_0921* in the F44, F53 and S80-9 genetic parasite background were monitored weekly by qRT-PCR analysis for a period of 8 weeks in several cell lines. Results indicated that the expression pattern of the *SVSP*s tested is largely stable and, at least *in vitro*, does not fluctuate significantly over the time period tested (data not shown).

In conclusion, while many *SVSP* genes appear to be expressed in different *T. parva*-transformed cell lines, the transcript levels of single *SVSP* genes can vary considerably depending on the parasite genotype. However, we found no evidence for a possible influence of the host cell background on differential *SVSP* mRNA expression between parasite clones with the same genotype.

### Detection of *T. parva SVSP* protein expression

**Figure 4:**
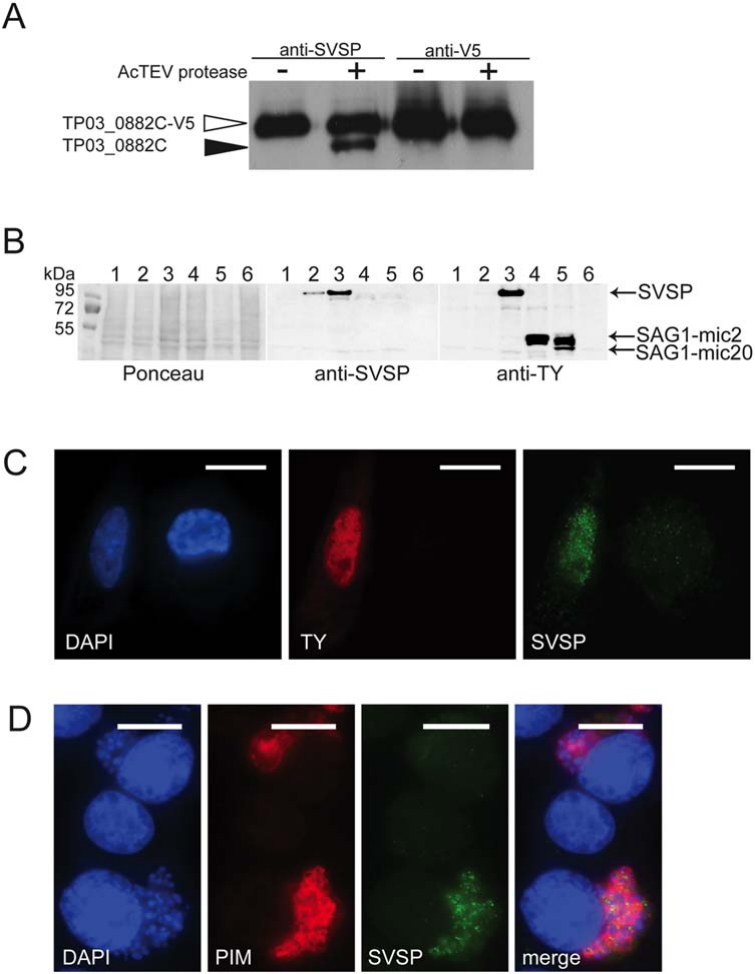
Detection of recombinant and parasite *SVSP* by immunoblot and immunofluorescence analysis. A. The C-terminal region of *SVSP TP03_0882* was expressed as a V5/His-epitope tagged protein in bacteria and subjected to immunoblot analysis using antibodies raised in rats against the same polypeptide (anti-*SVSP*) or anti-V5 antibodies. Open arrowhead indicates the uncleaved protein; closed arrowhead indicates C-terminal *TP03_0882* after removal of the V5/His epitope tag by AcTEV protease. B. BoMac cells were transfected with untagged and TY-tagged full length *TP03_0882* and analysed by immunoblot, using anti-*SVSP* or anti-TY antibodies. The position of *SVSP* and two TY-tagged control proteins SAG1-mic2 and SAG1-mic20 are indicated. A Ponceau-stained filter is added for loading control and also shows the position of the molecular weight markers. line 1: BoMac transfected with pmaxGFP (Amaxa); line2: BoMac transfected with pmaxCloning-TP03_0882; line 3: BoMac transfected with pmax-TP03_0882TY; line 4: BoMac transfected with pcDNA-SAG1-Ty-MIC2; line 5: BoMac transfected with pCAN-SAG1-Ty-MIC20; line 6: Whole cell lysate of *T. parva* (Muguga)- infected cells (TpMD409 CD8+ T cell). C. Detection of *TP03_0882 SVSP* expressed as a TY-tagged protein in BoMac cells. The nuclei of two cells are stained by DAPI. The cell on the left expresses *SVSP* as indicated by its reactivity with TY antibodies. The cell is also labelled after staining with anti-*SVSP* antibodies, showing nuclear localisation of the expressed protein. Scale bar: 20 μm. D. Cytospin preparations of *T. parva* (Muguga)-transformed cells (TpMD409 CD8+ T cells) mixed with uninfected bovine control BL20 cells were fixed in paraformaldehyde and analyzed by indirect immunofluorescence using anti-*SVSP* antibodies (green) and anti-PIM antibodies (red). Host and parasite nuclei were stained with DAPI (blue). The control cell in the middle does not harbour a parasite and is negative for PIM and *SVSP*. Both the cells at the top and the bottom are parasitised and thus labelled with anti-PIM. Only the parasite in the cell at the bottom, reacts with antibodies raised against *TP03_0882 SVSP* (green) partly revealing a vesicular staining pattern. Merge is an overlay of the three panels. Scale bar: 10 μm

To investigate whether *SVSP* expression can also be demonstrated at the protein level, we generated rat polyclonal antibodies directed against the C-terminal conserved domain of *SVSP TP03_0882*. Since the recombinant protein used for immunisation contained a V5/His epitope tag for detection and purification, the serum was also expected to contain antibodies against V5/His. To confirm that the antiserum contains antibodies against the *SVSP* backbone, the epitope tag was removed from the recombinant protein using AcTEV protease and the resulting protein fragments analysed by immunoblot. A lower molecular weight cleavage product lacking the V5 epitope was readily detectable confirming that antibodies specifically recognizing recombinant *SVSP* had been generated (Fig. 4A). This was also confirmed by the fact that antibodies reacted with untagged recombinant *TP03_0882* protein or with recombinant TY-tagged *TP03_0882* protein, but not with other TY-tagged proteins (Fig. 4B). *TP03_0882 SVSP* could not be detected in extracts prepared from *T. parva* (Muguga)-infected TpMD409 CD8+ T cells (Fig. 4B, line 6), most likely because only a minority of the parasitised cells express this protein (see below).

Using the anti-*SVSP* immune serum, we performed immunofluorescence analysis on BoMac cells expressing recombinant TY-tagged *TP03_0882* and also on *T. parva*-infected bovine leukocytes (TpMD409 CD8+ T cells). In BoMac cells, recombinant *TP03_0882* was readily detected, localising predominantly to the nucleus of the transfected cell. Fig. 4C shows one example of a BoMac-transfected cell in which the nucleus is strongly stained, except for the nucleoli. In other cells, the pattern of nuclear labelling could be very complex. The different nuclear localisation patterns will be presented separately below. In TpMD409 cells reactivity with schizonts could clearly be demonstrated (Fig. 4D). Co-staining with antibodies against the schizont surface protein PIM indicated that anti-*SVSP* antibodies predominantly recognized *SVSP*s located inside the schizont rather than on its surface. A marked dotted pattern, potentially representing the presence of *SVSP* in vesicles was observed. Anti- *TP03_0882* antibodies did not label all parasites. In cultures of *T. parva* (Muguga)-infected cells, antibodies recognised schizonts in less than 5% of the cells (see also Fig. 4D). One possibility is that not all parasite express TP03_0882. Alternatively, it is possible that in a large number of parasites the protein is expressed, but only at levels that are beyond detection by the anti-*SVSP* antibodies.

These results provide the first evidence for *SVSP* expression at the protein level by the *Theileria* parasite.

### The *SVSP* TP03_0882 targets nucleoli and other compartments within the nucleus of mammalian cells

**Figure 5:**
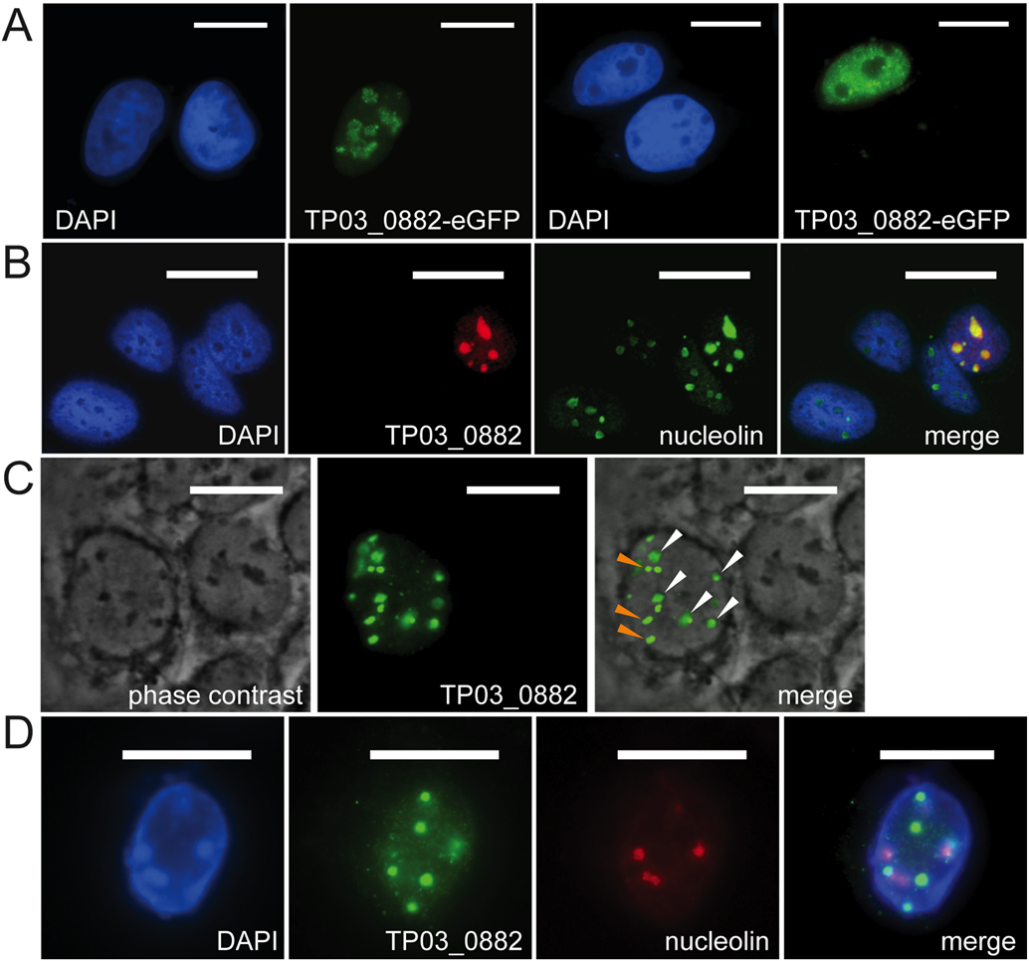
*SVSP TP03_0882* expressed in U2OS cells localises to different nuclear compartments. A. *TP03_0882* was expressed as eGFP fusion protein in U2OS cells. Cells growing on coverslips were fixed with paraformaldehyde. DNA was stained with DAPI (blue). Of the two cells shown in the panels on the left, one expresses *SVSP* which is localised to the nucleoli. The panels on the right show a cell with a nucleoplasmic *SVSP* localisation pattern. Scale bar: 20 μM. B. Cells transfected with a plasmid encoding V5-tagged *SVSP TP03_0882* were fixed with methanol and stained with antibodies directed against nucleolin (as nucleolar marker, green) or V5 (*SVSP*-expressing cell, red). The overlay (merge) reveals the colocalisation of *SVSP* and nucleolin in the nucleoli. Scale bar: 25 μm. C. In some transfected cells, *SVSP* (green) localises to nucleoli (white arrows) and other nuclear structures (orange arrows). In the left panel, nucleoli were visualized by phase contrast (dark areas). In the middle panel, *SVSP* is visualised using anti-V5 antibodies. Merge indicates the overlay of both panels. Scale bar: 20 μm. D. In some cells, *SVSP* (anti-V5, green) does not localise to the nucleoli (stained with anti-nucleolin, red) and is only found in dense nuclear bodies. Nuclei are stained blue (DAPI). Scale bar: 20 μm.

In order to test whether *SVSP*s, which are potentially secreted into the host cell cytoplasm, have a tropism for specific host cell structures or compartments, we expressed *TP03_0882* lacking the predicted N-terminal signal peptide in various mammalian cell lines and monitored localisation by immunofluorescence microscopy. Heterologous expression of a full-length *TP03_0882*-eGFP fusion protein in U2OS cells revealed that the protein almost exclusively localised to the nucleus (Fig. 5A). Within the nucleus different localisation patterns could be observed. In some cells, *TP03_0882*-eGFP accumulated mainly in compartments resembling the nucleoli (Fig. 5A, left panels), whereas in other cells the protein was found predominantly in the nucleoplasm while nucleoli were clearly excluded (Fig. 5A, right panels). The same localisation patterns were found upon expression of *TP03_0882* containing a C-terminal V5 epitope tag, indicating that the nuclear accumulation is not an artefact caused by the eGFP fusion portion of the polypeptide. In addition, similar patterns were observed when anti-*SVSP* antibodies were used (not shown). To confirm the nucleolar localisation counterstaining with anti-nucleolin was performed. Fig. 5B shows *TP03_0882-V5* colocalisation with nuclear structures clearly recognised by anti-nucleolin antibodies. In a number of cells, *TP03_0882-V5*, in addition to targeting nucleoli, was also found to localise to other nuclear structures (Fig. 5C). While a large proportion of *TP03_0882-V5* could be found in the nucleoli (recognizable as dark-staining areas by phase contrast microscopy), other, clearly defined spherical structures of unknown origin were also strongly labelled. Finally, in other cells, *TP03_0882-V5* failed to associate or associated only weakly with nucleoli and predominantly the spherical nuclear structures were labelled (Fig 5D). Dual labelling with anti-V5, visualising *SVSP*, and antibodies that recognise telomeres did not reveal any colocalisation (not shown). Similar localisation patterns were detected in all transfected cell lines examined, including COS-7, HeLa and *T. annulata*-infected macrophages (not shown). When *TP03_0882* constructs lacking the N-terminal 368 aa were expressed, nuclear localisation still occurred, indication that the QP-rich variable region does not contribute to nuclear translocation (data not shown).

### Functional analysis of potential, parasite-encoded nuclear localization signals

Importation of proteins into the nucleus is classically mediated by recognition of nuclear localisation signals (NLS), which fall into two major classes. Analysis of the *TP03_0882* amino acid sequence using the PsortII [34] and NucPred [35] prediction programs revealed the presence of two potential NLS. The NucPred algorithm predicted an SV40 large tumor antigen-type NLS (designated NLS1, aa 340–351; see Fig. 1). A bipartite NLS (aa 394–410; NLS2) was detected using the PSORTII algorithm. The presence of NLS is not specific for this individual gene, since using both algorithms, putative nuclear import motifs could be found in 78% of the *T. parva SVSP* family members (Table S2).

**Figure 6:**
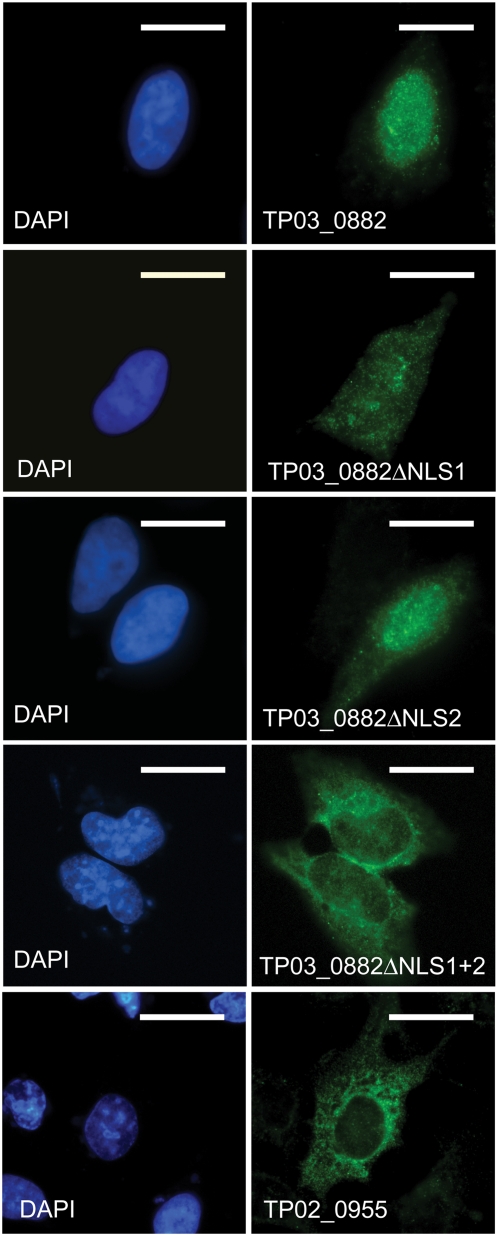
Identification of two functional NLS in SVSP TP03_0882. U2OS cells were transfected with plasmid constructs encoding V5-tagged forms of TP03_0882 containing both NLS (TP03_0882), lacking NLS1 (TP03_0882ΔNLS1), lacking NLS2 (TP03_0882ΔNLS2) or both NLS (TP03_0882ΔNLS1+2). In addition, cells were also transfected with a plasmid encoding *TP02_0995*, an *SVSP* predicted not to contain a NLS. Cells were fixed in methanol and *SVSP* localisation monitored using anti-V5 antibodies (green). DNA was stained with DAPI (blue).

To investigate to what extent these NLS contribute to targeting *TP03_0882* to the nucleus, constructs lacking either NLS1 (pTP03_0882ΔNLS1), NLS2 (pTP03_0882ΔNLS2) or both (pTP03_0882ΔNLS1+2) were generated and the sub-cellular localisation of the V5-epitope tagged proteins monitored by immunofluorescence microscopy in transfected U2OS cells. Upon deletion of NLS1, the protein still translocated to the nucleus, but, in contrast to wild-type *TP03_0882-V5*, a large proportion of the protein was retained in the cytoplasm (Fig. 6). Similar observations were made when NLS2 was deleted (pTP03_0882ΔNLS2). Upon removal of both nuclear localisation motifs (pTP03_0882ΔNLS1+2), however, the protein was to a large extent excluded from the nucleus, indicating that NLS1 and NLS2 can function independently, but that both motifs are required for optimal and efficient import of *TP03_0882* into the nucleus. For a minority of other members of the *SVSP* family such as *TP02_0955*, PSORTII or NucPred failed to identify aa sequences predicting an NLS. When epitope-tagged *TP02_0955-V5* was expressed in U2OS cells, the protein remained almost exclusively in the cytoplasm, similar to TP03_0882ΔNLS1+2.

## Discussion

This study presents an initial characterisation of the expression of the *T. parva SVSP* family, the largest gene family identified in *Theileria* so far [24], [25]. We found that in three different cell lines infected with cloned *T. parva* (Marikebuni) parasites, transcripts corresponding to more than 65% of *SVSP* genes are expressed simultaneously. This is in agreement with the findings of a whole-transcriptome analysis by MPSS, performed on a *T. parva* (Muguga)-infected cell line [28] which showed that *SVSP* mRNA can be detected at low to medium levels and also that genes with similar expression levels are distributed over the eight subtelomeres showing no clustering at specific locations.

While *SVSP* transcript levels in different infected cell lines appear to be largely comparable, specific *SVSP* genes exhibiting differential expression were identified. We investigated the effects of three variables that could conceivably have an impact on *SVSP* expression levels: the individual animal background the infected lymphocytes were derived from, the host cell type (T or B lymphocyte) and the parasite genotype. The latter was found to be the key determinant of differential expression between copies within the gene family. Cell lines containing clonal parasite genotypes showed *SVSP* mRNA expression patterns that differed between the clones, but were not affected by host cell type or animal background (Figs. S1 and 2). The most pronounced differences were found for the *SVSP* genes *TP03_0869* and *TP04_0921*, located on the reverse strands of chromosomes 3 and 4. When compared to other *SVSP*s, the only obvious difference is that in *TP04_0921*, the QP-rich region is lacking, which is not the case for *TP03_0869*. The absence of the QP-rich region has also been observed for a few other *SVSP*s, however, and is unlikely to explain the differences in mRNA expression. Like most *SVSP*s, *TP03_0869* and *TP04_0921* contain a predicted signal peptide potentially allowing access to the secretory pathway, two FAINT domains in the C-terminal region and no predicted transmembrane domains, which is consistent with secretion. While *TP03_0869* has no special position in the *SVSP* array, *TP04_0921* is the most telomere-proximal *SVSP* gene on the reverse strand of chromosome 4. Although no absolute quantification was carried out, qRT-PCR experiments revealed that, compared to all other *SVSP* genes, the C_t_ values of *TP03_0869* and *TP04_0921* are much higher in the cell line 951T_F44, compared to other cell lines. This suggests that transcription of the two genes is markedly downregulated in 951T_F44 rather than upregulated in the other cell lines.

The sequences of *TP03_0869*, *TP03_0883* and *TP04_0921* (showing the highest differences in transcript levels between the different parasite genotypes) and the intergenic region between *TP04_0928* an *TP04_0921* are conserved between the different parasite genotypes, indicating that differences in mRNA expression are not caused by polymorphism. Polymorphism is not unexpected as it is prevalent in clusters of neighbouring genes of the *Sfi*I fragment related family which are also subtelomeric [33].

We also established that mRNA expression patterns appear to be fairly stable as mRNA expression of three *SVSP* genes tested in different cell lines remained largely constant over an observation period of up to 8 weeks. Whether *SVSP* expression in cloned parasites would also be constant *in vivo*, represents an interesting issue for future investigation.

It will be of interest to determine whether individual parasites express single *SVSP* mRNAs, or whether a selection of different *SVSP* genes can be expressed by a single schizont. Also, since schizonts are multinuclear, it remains to be established whether individual *SVSP* genes are transcribed in one or several schizont nuclei within a single infected cell.

Raising antibodies to *SVSP*s proved very difficult and required multiple attempts in different animal species. This is in marked contrast to the schizont surface protein PIM, which also has a QP-rich domain and –as indicated by its name-is very immunogenic, both in cattle and mice. The observation that polyclonal antibodies directed against the *SVSP TP03_0882* show strong staining in only a small number of parasitized cells indicates that not all parasites express the protein or that expression levels vary considerable between individual parasites. It also suggests that antibodies directed against the structurally conserved *SVSP* C-terminal domain encoded by *TP03_0882* probably do not generally cross-react with other *SVSP*s. At this stage we cannot rule out the possibility that *SVSP TP03_0882* is expressed at low levels below the detection threshold of immunofluorescence analysis in additional cells, or that more than one *SVSP* is expressed per parasite. Similar results were obtained when other cell lines were investigated, suggesting that restricted and/or strongly varying levels of expression is a typical feature of *SVSP*s.

Immunofluorescence analysis showed a clear accumulation of *TP03_0882 SVSP* in vesicular structures, supporting the notion that *SVSP*s are incorporated into the parasite secretory pathway. Transfection studies in mammalian cells also confirm that the predicted NLS could help target *SVSP*s to the host cell nucleus. Nevertheless, under the conditions used, the anti-*SVSP* antibodies that we generated did not allow us to confirm that *SVSP*s are indeed secreted into the host-cell cytoplasm and translocate to the host cell nucleus. It is possible, that only small amounts of *SVSP* are secreted and that our detection tools are not sensitive enough. Alternatively, it is possible that secreted *SVSP*s are rapidly degraded resulting in low steady state levels of protein.

*SVSP*s are a unique family with no known orthologues in genera other than *Theileria*. The function of both the unstructured N-terminal QP-rich region and the conserved C-terminal region containing the FAINT domains are unknown. The fact that the QP-rich region of the protein is lacking in some *SVSP*s suggests that this variable region is not indispensable for protein function. It also indirectly supports the notion that the conserved C-terminal domain, which also contains the NLS, could be the main contributor to the–still unknown-biological function(s) of *SVSP*s. It is intriguing to note that *TP03_0882* localises to defined nuclear structures when expressed in mammalian cells. Nuclear localisation has also been observed for other *Theileria* proteins such as TashAT and SuAT proteins [15], [16], [36], [37]. Ectopically expressed *TP03_0882* was frequently found to localise to the nucleoli. The nucleolus is a multifunctional subnuclear compartment that is best known as the site where ribosome biogenesis takes place. However, different studies also point towards an involvement of nucleoli in processes linked to cell cycle control, senescence, DNA replication and repair, and the regulation of stress responses (reviewed in [38]). The possibility that *Theileria* proteins might target the host cell nucleolus is interesting in view of the fact that several viral proteins, including the Rex protein of the transforming human T-lymphotropic virus (HTLV-1) [39] and a portion of the adeno-associated virus protein Rep [40] can also be found in nucleoli. *TP03_0882* did not localise to nucleoli in all cells, however, and was sometimes found in association with other nuclear structures. Each cell cycle, the nucleolus undergoes extensive reorganisation and the nucleolar proteome is also subject to dynamic changes dependent on cellular conditions [41]. Such changes might represent a factor in determining the pattern of *TP03_0882* localisation to the nucleolus. Alternatively, nucleolar targeting of *TP03_0882* might be subject to cell cycle-dependent changes in post-translational modifications (see [38] and references therein).

Whether *SVSP* genes are expressed in a mutually exclusive manner as has been described for *var* genes of *P. falciparum* remains to be established. *Var* genes, many of which are also subtelomeric, encode PfEMP1, a surface protein involved in adherence of infected erythrocytes to epithelial cells or to other erythrocytes. Only one PfEMP1 is expressed at the time and expression involves multiple layers of control involving chromatin modifications, nuclear positioning, and promoter-intron interactions [2], [42], [43]. Switching PfEMP1 expression allows intra-erythrocytic *Plasmodium* to avoid clearance by the host spleen. Rather than being expressed on the surface, *SVSP*s are predicted to be secreted into the host cell cytoplasm. Proteins secreted by the *Theileria* schizont can access the MHC class I–CD8+ T cell surveillance system which plays a central role in immunity to *T. parva*. However, despite the theoretical abundance of potential candidates [21], in individual animals, this immunity is tightly focused on only a few immunodominant MHC–peptide epitope combinations (reviewed by [19], [44] leading to a limited cross-protection between different strains. At this stage, it is not known how efficiently *SVSP*s are processed for presentation through the MHC class I pathway. Nevertheless, assuming each individual *SVSP* released into the host cell cytoplasm could potentially provide epitopes capable of priming protective cytotoxic responses, the simultaneous release of a large number of similar *SVSP* proteins would by definition increase the chances of detection and elimination of parasitised cells. Our initial findings using antibodies directed against *TP03_0882* suggest that this specific *SVSP* is expressed by only a small fraction of the parasites. Although we cannot exclude the concurrent expression by the same parasite of additional *SVSP*s, our initial findings indicate that the expression of individual *SVSP*s may be restricted. It has been shown that aminopeptidases in the endoplasmic reticulum that process antigenic precursors generated by proteasomal degradation, thus generating peptides suitable for presentation by MHC class I molecules, fail to trim prolines that flank the NH2-termini of these precursors [45]. To what extent prolines in the QP-rich region of *SVSP*s might interfere with efficient peptide generation remains to be established.

In conclusion, using cell lines derived from individual *T. parva* clones, we provide a first characterisation of the expression pattern of members of this large and intriguing *T. parva* gene family. *SVSP* genes are also found in the closely related parasite *T. annulata*, potentially reflecting a conserved biological role. The tropism *SVSP*s show for different sub-nuclear compartments of the mammalian cell could provide important clues concerning their biological function.

## Materials and Methods

### Cell lines and parasites

Cloned *T. parva*-infected cell lines used for analysis of *SVSP*-transcript patterns (Table 2) were generated and cultured as described [29], [30], [31]. Parasite genotypes A3, F31, F44, F53 are referred to as 72-01, 72-30, 72-27 and 72-29 respectively in [31] and originate from stabilate 72. The cell line 951T_I8 was generated from a different *T. parva* (Marikebuni) stabilate originally obtained from Kenya Agricultural Research Institute (KARI). The *T. parva* (Muguga) clone 80.9 was newly generated and is not published. Parasite genotypes were determined with *T. parva*-specific satellite markers [31], [46] and host cells were typed using flow cytometry. Native *SVSP*s were detected in *T. parva* (Muguga)-infected CD8+ T cells [47]. Expression of recombinant *SVSP* was analyzed in U2OS and BoMac cells cultured under standard conditions.

### Microarray and quantitative real-time PCR analysis

Total RNA was extracted from schizont-infected cells using the RNeasy Mini Kit according to the manufacturer's protocol (QIAGEN). Each infected cell line was represented by two biological replicates. RNA was treated on-column with RNase-free DNase (QIAGEN) to remove contaminating genomic DNA. Conventional end-point PCRs were performed with *T. parva* actin (5′-CGGGATCCGACCCACAATGTGCCA-3′ and 5′-CGGAATTCACGGTGCACAATATT-3′) and PIM primers (5′-CGAAGCTTGAAAACATGAAGATCTTTCCC-3′ and 5′-TACGCGGCCGCTTAACAACAATCTTCGTTAATGCG-3′) using GoTaq DNA Polymerase (Promega) to detect contamination with genomic DNA. The RNA was considered ‘DNA-free’ when no amplification product was detectable after 30 cycles.

For microarray analysis, a direct two-colour experimental design was used to compare parasite transcripts levels using 70-mer oligonucleotide microarrays containing 4060 *T. parva* genes (Kang'a *et al.*, in preparation). Hybridisations were performed using 15–20 μg of Cy-dye labelled amplified-RNA (aRNA) from each infected cell line. Briefly, cDNA targets were first prepared from 5 μg of total parasite RNA extracted from each *T. parva*-infected cell line by reverse transcription followed by *in vitro* transcription with simultaneous incorporation of amino-allyl dUTP using the Aminoallyl Message Amp RNA amplification kit (Ambion Inc., Austin TX) according to manufacturer's instructions. The resulting aRNA was fluorescently labelled using Cy3 and Cy5 dyes through coupling with amino-allyl dUTP in 0.1M Sodium Carbonate buffer. The labelled targets were used to hybridise the 70-mer oligonucleotide probes on the glass slides by incubation for 16 h at 42°C in a hybridisation buffer consisting of 50% formamide, 5× SSC, 0.1% SDS, and 0.6 μg μl^−1^ salmon sperm DNA. Flip-dye hybridisations were performed by reversing Cy-dyes for each pair of hybridised parasite aRNA. Following a 16 h incubation, slides were sequentially washed in 2× SSC, 0.1% SDS for 5 min at 42°C, 0.1× SSC, 0.1% SDS for 5 min at room temperature, 0.1× SSC for 5 min at room temperature and dried by centrifugation. Scanning and data extraction was performed using GenePix® 4000B Axon scanner and Genepix 5.0 software respectively (Axon Instruments, Union City CA).

Data were analyzed using the *marray* packages from the Bioconductor site (www.bioconductor.org; [48]). Following data normalisation, executed in two steps, first using within-slide loess normalisation followed by quantile between–slide normalisation, we employed linear models [49] to rank the differentially expressed genes in order of evidence (i.e., *P* value) of differentially expressed genes. Briefly, the function *lmFit* in *limma* accepts a model matrix, which describes the experimental design and produces an output object of class *MarrayLM*, which stores the fitted model information for each gene. The fitted model object is further processed by the *eBayes* function to produce empirical Bayes test statistics for each gene, including moderated *t*-statistics, *P*-values and log-odds of differential expression. Genes with P<0.05 and with log2-fold change (M) value of <−0.9 or >0.9 (representing approximately 2 fold change in transcript abundance) were considered as significantly differentially transcribed. Since each gene on the array was printed in quadruplicate, the final list of unique genes was generated by calculating the medians of in-slide replicates for genes that met these criteria. Accession Nr: GEO14093.

For quantitative real-time (qRT-PCR), first strand cDNA synthesis was performed from 2 μg total RNA using RNase H deficient M-MLV reverse transcriptase and 1 μg oligo(dT) primer in the presence of RNasin Ribonuclease inhibitor as described by the manufacturer (Promega). Reverse transcriptase was heat-inactivated for 5 min at 80°C and to verify the generation of cDNA products, end-point PCR reactions were performed as described above.

Transcript levels of selected *SVSP*s were determined by qRT-PCR using SYBR green dye technology (Applied Biosystems). The Primer Express Software v.2.0. (Applied Biosystems) was used to design the primers shown in Table 1. The oligonucleotide sequences were analyzed for specificity using BLAST searches (www.ncbi.nlm.nih.gov/BLAST). Since the housekeeping genes fructose bisphosphate aldolase (*FBA*, *TP01_0101*) and actin (*TP02_0903*) are highly conserved in a wide range of species, coding sequences of the *T. parva* and bovine homologues were aligned using CLUSTALW at EMBnet-CH and sequences with low similarity were selected manually. Primer pairs for these genes were subsequently designed with the Primer3 software [50]. For the *SVSP TP04_0007*, no sequences were found which do not at the same time occur in at least one other *SVSP* gene. For this gene, primers were designed such that the forward primer does not cross-hybridize with the same genes as the reverse primer. All oligonucleotides were synthesized by Microsynth (Balgach, Switzerland). Since the *SVSP* genes contain highly conserved regions, primer pairs were first tested and only primer pairs were used for which single melting peaks were obtained.

**Table 1:** Primer pairs used for quantitative RT-PCR

Locus Name	Forward Primer	Reverse Primer
TP01_0004	tgagatgtgcggacaaacca	gacccacactcttggcagttg
TP02_0953	gttatggaacatgggcttgga	tcatgtggtgttggcggtata
TP02_0954	aaggcatcctgtgccacatc	gcctgtggtggctgataaaca
TP03_0869	tgtttaaaaatgatggtgaaggaaatt	agtcttttccagacatttcaaccaa
TP03_0877	cactcggatcctttaaatatgtattgc	cccataccatcagaccatcaact
TP03_0882	ccatcaagtcaaccaaggtatcag	ctctattggttgattaggccgataa
TP03_0883	aactgactttacgattccagactatgtc	ttccctccaatatcttgtgtgaac
TP03_0887	ttccggattatcacccaggtt	gaaatgtgtaaataggcggaacatt
TP03_0890	gggcctcaagaatatcctggat	atgttgataaccaggatcgtagacaa
TP04_0007	tggaaaactggtagaaatgataaatg	ttccatcttcatggaggtagataat
TP04_0018	ccccagaccaatatacagtccaa	tgtagcgaaaaccaccgtatga
TP04_0921	cataataattggatgtataaaatgctctga	cctgacttatcaccaggcttatca

**Table 2:** *T. parva*-infected cell lines established by transformation with different *T. parva* clones.

Cell line	Parasite isolate	Parasite genotype	Animal	Host cell type	Reference
211T_A3 (211-72-29)	Marikebuni	A3	211	CD4+	[30]
211B_A3 (211-72-33)	Marikebuni	A3	211	B cell	[30]
951T_F44 (951-F44)	Marikebuni	F44	211	CD4+	[31]
951B_A3 (951-A3)	Marikebuni	A3	951	B cell	[31]
951T_I8 (951-I8)	Marikebuni	I8	951	γδ	
951T_F31 (951-F31)	Marikebuni	F31	951	γδ	[31]
951T_F53 (951-F53)	Marikebuni	F53	951	CD4+	[31]
951T_S80.9 (951-S80.9)	Muguga	80-9	951	CD4+	none
TpMD409	Muguga		D(409)	CD8+	[47]

The efficiency of amplification was assessed for each primer pair by generating standard curves plotted as C_t_ versus log concentration. Only primers and primer concentrations yielding a slope between −3.2 and −3.6 were used.

All qRT-PCR reactions were performed in duplicates in a total volume of 25 μl containing Power SYBR Green Master Mix (Applied Biosystems) and 250 or 500 nM of each primer (depending on reaction efficiency). For quantification of transcript levels in the different cell lines, 40 ng cDNA were used per reaction. The assays were carried out with the ABI Prism 7700 Sequence Detector System and associated Sequence Detection Software v.1.9.1 (Applied Biosystems) following the manufacturer's guidelines. For each cDNA sample, a reaction was carried out with the corresponding amount of input RNA without reverse transcription and for each reaction a melting temperature profile was generated to ensure the amplification of a single product. To detect unspecific amplification of bovine DNA, control reactions were performed using cDNA of an uninfected bovine cell line (BL20) as template.

For final quantification of the relative transcript levels, the comparative C_t_ method was applied using fructose-bisphosphate aldolase (*TP01_0101*) as an endogenous control and the sample with the lowest expression level of the respective gene as calibrator.

### Generation of anti-SVSP antibodies

The C-terminal part of *TP03_0882* (bp 1075–1821, AA 359–607) was amplified with primers 5′-CACCCATGAAACTACTCAAAAATC-3′ (sense) and 5′-TTATCTATATTTGGCTTTTATTAAATT-3′ (antisense) using pTP03_0882 as template. The PCR fragment was directly cloned into pET151/D-TOPO (Invitrogen) a bacterial expression vector resulting in pET151-TP03_0882C. TP03_0882C protein was over-expressed in *E. coli* BL21 star cells (Invitrogen) and the 6xHis tagged protein was purified over Protino Ni 150 columns (Machery Nagel, #745 100.10). The purified protein was mixed with GERBU adjuvant (GERBU Biotechnik GmbH, Gaiberg, Germany) and rats were immunised. The diluted immune serum was used for immunofluorescence and Western blot analysis. These polyclonal antibodies are not necessarily mono-specific for *TP03_0882* and may recognise more than one *SVSP* protein, as the protein family has conserved C-terminal ends and certain epitopes may therefore be shared with other *SVSP*s. In order to cleave the V5/His–tag from recombinant TP03_0882C-V5 40 μg of purified protein was incubated for 3 h at 30°C in the presence of 20 U of AcTEV protease, 1 mM DTT and 1×TEV buffer (Invitrogen).

### Recombinant *SVSP* constructs

Different versions of *TP03_0882* and *TP02_0995* were expressed as recombinant proteins in mammalian cells either tagged with V5 or TY epitopes or as eGFP fusion proteins. All constructs expressed in mammalian cells lack the predicted signal peptide for secretion. To generate pTP03_0882, PCR was performed with the primers 5′-AT*AAGCTT*ATGCCTGATCAACCTGCTGATGAT-3′ (sense) and 5′-GC*CTCGAG*TCTATATTTGGCTTTTATTAAATT-3′ (antisense) using genomic DNA from the T-cell line 803 containing *T. parva* (Muguga) as template. Since in preliminary experiments we had initially isolated an incomplete cDNA clone corresponding to *TP03_0884,* the DNA sequence of the primers was based on the *TP03_0884* gene locus, also a member of the *SVSP* gene family. As all *SVSP* family members have very conserved 5′- and 3′-ends in our PCR reaction *TP03_0882* was amplified instead, resulting in a protein with the following changes compared to the original *TP03_0882* protein sequence published in the TIGR *T. parva* genome database: S30A, E31D, T607R, all conservative amino acid changes, introduced by using the above described primers. We found two additional changes in our PCR fragment by comparing the DNA sequence to the *TP03_0882* sequence in the TIGR database. Both changes are A to G transitions which lead to the following amino acid changes: D45G and T158A. The PCR product was directly cloned into pCR®2.1-TOPO (Invitrogen) resulting in pTOPO-TP03_0882 and from there sub-cloned into the mammalian expression vector pcDNA3.1/V5-HisA (Invitrogen) using the restriction enzymes *Eco*RI and *Hin*dIII. The final construct produces a truncated version of *TP03_0882* from amino acid 26 to 607 containing the above-indicated changes and is referred as *TP03_0882* in this paper. pTP03_0882 was used as template for constructing three plasmids coding for *TP03_0882* versions lacking either one of the two putative NLS or both. To construct pTP03_0882ΔNLS1 the following primers were used to amplify the N-terminal part (bp 26–1098) of *TP03_0882*: 5′-TAATACGACTCACTATAGG-3′ (sense), 5′-CTGT*CCATGG*AGTTGATTTTTGAGTAGTTTC-3′ (antisense) and the two following primers were used to amplify the C-terminal part (bp 1144–1821): 5′-GCTG*CCATGG*GATCAAACTGAGAAGAAAGATG (sense) and 5′-GCCTCGAGTCTATATTTGGCTTTTATTAAATT (antisense). The amplicon encoding the N-terminal section was digested with the restriction enzymes *Eco*RI and *Nco*I, the fragment corresponding to the C-terminal was digested with *Nco*I and *Xho*I. Both fragments were ligated simultaneously into pcDNA3.1/V5-His linearized with *Eco*RI and *Xho*I. The same cloning strategy was used to construct pTP03_0882ΔNLS1+2. The same N-terminal part (bp 26–1098) as used for construction of pTP03_0882ΔNLS1 cut with *Eco*RI and *Nco*I was ligated with the C-terminal part (bp 1231–1821) amplified with the primers 5′-GCATG*CCATGG*AATTATCTGGGAAAGCTTG-3′ (sense) and 5′-GCCTCGAGTCTATATTTGGCTTTTATTAAATT-3′ (antisense) and cut with *Nco*I and *Xho*I into *Eco*RI/*Xho*I linearized pcDNA3.1/V5-His. The first TP03_0882ΔNLS2 construct was made as eGFP fusion protein in the vector peGFP-N3 (BD Biosciences Clontech). Amplification of the N-terminal part (bp 26–1179) was performed with the primers 5′-TAATACGACTCACTATAGG-3′ (sense) and 5′-GCATG*CCATGG*AACAGATTCTTCACCATC-3′ (antisense). The C-terminal part (bp 1231–1821) was amplified with the primers 5′-GCATG*CCATGG*AATTATCTGGGAAAGCTTG-3′ (sense) and 5′-CGC*GGATCC*GGTATATTTGGCTTTTATTAAATTT-3′ (antisense). The PCR product of the N-terminal part was cut with *Eco*RI and *Nco*I, the PCR product of the C-terminal part was cut with *Nco*I and *Bam*HI, both fragments were ligated simultaneously to *Eco*RI/*Bam*HI linearised peGFP-N3 to generate pTP03_0882ΔNLS2-EGFP. This vector was used to generate pTP03_0882ΔNLS2. pTP03_0882ΔNLS2-EGFP was cut with *Hin*dIII to cut out a 1176 bp fragment harbouring the ΔNLS2 truncation. This ΔNLS2 *Hin*dIII fragment was ligated into *Hin*dIII cut pTP03_0882 replacing the N-terminal part containing NLS2. The resulting vector is called pTP03_0882ΔNLS2. For expression of the TP03_0882 as a GFP fusion protein, the cDNA was subcloned from pTOPO-TP03_0882 using the restriction enzymes *Sac*I and *Apa*I and ligated into pEGFP-N3 cut with the same enzymes. The resulting vector is called pTP03_0882-eGFP. We also cloned a gene of the *SVSP* family, which does not originally contain a NLS, *TP02_0955* a single exon gene. The gene lacking the N-terminal region coding for the signal peptide for secretion (bp 58–1683) was amplified using the primers 5′-AAT*GAATTC*ACCACCATGGATAGATATCCCTATCAC-3′ (sense) and 5′-TGAC*CTCGAG*ATAATCTTTTACATTCTTTCTA-3′ using genomic DNA of the T-cell line 951-F31 harbouring *T. parva* (Marikebuni) as template. The PCR product was digested with the restriction enzymes *Eco*RI and *Xho*I and ligated into *Eco*RI/*Xho*I linearized pcDNA3.1/V5-His for expression in mammalian cells resulting in pTP02_0955. All DNA fragments amplified by PCR were sequenced (Microsynth AG, Balgach, Switzerland) to verify the correct DNA sequence. To bypass problems of low expression levels with the pcDNA constructs, most of the *SVSP* constructs were subcloned into the pmaxCloning expression vector (Amaxa) using standard protocols, resulting in much higher *SVSP* expression levels in mammalian cells.

### Immunofluorescence analysis

For detection of endogenous *SVS* proteins, cytospins with *T. parva-*infected T-cells (TpM D409) were performed. All the following steps were carried out at room temperature. Cells were fixed in 4% paraformaldehyde for 20 min and subsequently permeabilized in 0.2% Triton X-100 (prepared in PBS) for 5 min. Nonspecific binding was blocked with PBS containing 10% heat inactivated FCS (FCS/PBS) for 10 min. Anti-*SVSP* immune serum was diluted 1∶200 in FCS/PBS and applied for 1 h. Cells were washed three times with PBS and incubated with secondary antibody Alexa488-conjugated goat anti-rat antibody (1∶1000; Invitrogen, Molecular Probes A-11006) for 1 h. Cells were washed five times with PBS and then incubated with anti-PIM 40.2 antibodies (1∶1000; IL-S40.2, International Livestock Research Institute, Nairobi, Kenia) in FCS/PBS for 1 h to visualize the parasite surface. Cells were washed again three times with PBS and incubated with secondary antibody Texas Red-conjugated goat anti-mouse antibody (1∶800; Invitrogen, Molecular Probes T862) for 1 h in FCS/PBS. DNA was stained for 1 min with DAPI (4′,6-diamidino-2-phenylindole, dihydrochloride; Molecular Probes, D-21490) at a concentration of 1 μg ml^−1^ in PBS. Finally, the slides were covered with a coverslip using anti-fading mounting reagent (Vectashield). For the overexpression studies, U2OS cells were grown on glass coverslips and plasmid DNA was transfected with lipofectamine (Invitrogen life technologies) according to the manufacturer's protocol and cells were analyzed 24 h post transfection. For immunofluorescence the same protocol was applied as for detection of endogenous *SVSP*s but cells were mainly fixed in cold (−20°C) methanol for 10 min and primary antibodies raised in mouse or rabbit could be applied together as well as secondary anti-mouse and anti-rabbit antibodies. Primary antibodies were diluted as followed: mouse anti-V5 antibody (1∶500; Invitrogen life technologies R960-25), to visualize nucleoli rabbit anti-C23 (H259) antibodies were used (1∶200; Santa Cruz sc-13057). Secondary antibodies were used at the following dilutions: Alexa488-conjugated goat anti-rabbit antibody (1∶1500; Molecular Probes A-11034), Alexa488 conjugated goat anti-mouse antibody (1∶1500; Molecular Probes A-11029), Texas Red-conjugated goat anti-mouse antibody (1∶800; Invitrogen, Molecular Probes T862). To visualize eGFP fusion proteins cells were fixed in 4% paraformaldehyde for 10 min at room temperature. DNA was stained with Dapi and coverslips were mounted onto slides using mounting reagent (Vectashield). All samples were analyzed using a Nikon Eclipse fluorescence microscope 80i and digital images were generated and processed using the Openlab 3.1.5 software.

### Immunoblot analysis

Whole cell lysates of *T. parva*-infected TpM(409) CD8+T-cells or transfected BoMac cells were prepared in 1× Lämmli-buffer containing 4% β-mercaptoethanol. The lysates were sonicated and insoluble cell debris was removed by centrifugation at 23'000×g for 10 min at 4°C. Samples were stored at −20°C and aliquots of 20 μl cell lysate (2×10^5^ cells) were used for Western blot analysis. Immunoblot analysis was performed according to standard procedures. Samples were boiled for 5 min before loading onto a 10% SDS-polyacrylamide gel. After blotting, the nitrocellulose membranes were blocked for 1 h with 5% milk-TBST (10 mM Tris, pH 8.0; 150 mM NaCl; 0.05% Tween-20). The membranes were incubated in the same blocking buffer containing rat anti-*SVSP* immune serum diluted 1∶500, mouse anti-TY antibodies 1∶2000 (D. Soldati, Geneva, Switzerland) or mouse anti-V5 antibodies (P/N 46-0705, Invitrogen) 1:2000 over night at 4°C. Membranes were washed 3× for 5 min in TBST and then incubated for 1 h at room temperature in 5% milk-TBST containing secondary antibodies: horseradish peroxidase-conjugated goat anti-rat antibody (SC-2006, Santa Cruz Biotechnology,) or horseradish peroxidase-conjugated rabbit anti-mouse antibody (P0260, Dako) both diluted 1∶2000. Membranes were washed 3× for 5 min with TBST and once for 5 min with TBS (10 mM Tris, pH 8.0, 150 mM NaCl). For visualisation of the signal in the FUJIFILM LAS-3000 system, the ECL detection system was used according the manufacturer's instructions (Amersham Biosciences).

## Supporting Information

Figure S1 Schematic presentation (adapted from (Bishop *et al*., 2005)) depicting the organisation of *SVSP* genes at the subtelomeres of the four *T. parva* chromosomes. *SVSP* genes are shown in red and the numbers represent the original numbering allocated to each of the proteins encoded by the *T. parva* (Muguga) *SVSP* genes (Gardner *et al*., 2005). Sfi subtelomeric related family genes are marked in blue. Genes unrelated to these two families are coloured green and telomeres are grey. Asterisks indicate *SVSP* transcript levels as defined by Bishop et al. (Bishop *et al*., 2005) by MPSS analysis of a cell line established by infection with a *T. parva* (Muguga) clone. One asterisk reflects 4 to 99 transcripts per million; two asterisks indicates 100 to 999 transcripts per million; the absence of an asterisk indicates that no transcripts were detected. Microarray (MA, circles) or quantitative real-time PCR (qRT, squares) analyses were also carried out to investigate mRNA expression in cell lines established by infection with different *T. parva* (Marikebuni) clones (see results section). Pairs of genes numbered 1-5 are identical. The absence of circles or squares means that these genes were not analysed. Open circles or squares indicate genes for which no transcripts were detected. Red circles and squares reflect genes for which no difference in mRNA expression was found. Red circles with a white centre indicate that one or both identical genes are expressed. Genes found to be differentially expressed using MA or qRT are marked by green circles or squares, respectively.
(0.57 MB EPS)

Table S1 Microarray analysis of *SVSP* expression patterns in three cell lines
(0.13 MB DOC)

Table S2 List of *T. parva SVSP* genes, indicating the presence or absence of a signal peptide and/or nuclear localisation signal
(0.09 MB DOC)
