# Supplementary material for: Expression Analysis of the Theileria parva Subtelomere-Encoded Variable Secreted Protein Gene Family
Source: PLoS One. 2009 Mar 27;4(3):e4839. doi: 10.1371/journal.pone.0004839 (PMC2657828; doi:10.1371/journal.pone.0004839)
Supplement: Table S1 — Microarray analysis of SVSP expression patterns in three cell lines (0.13 MB DOC) [file pone.0004839.s002.doc]

**Table S1**

Microarray analysis of *SVSP* expression

|  |  |  | MICROARRAY HYBRIDIZATION RESULTS | | |
| --- | --- | --- | --- | --- | --- |
|  | **Locus** |  | **211T_A3 vs 211B_A3** | **951T_F44 vs 211B_A3** | **951T_F44 vs 211T_A3** |
| **Chromosome 1** | TP01_0004 |  | P | P | 1.8 |
|  | TP01_0005 |  | P | P | P |
|  | TP01_0006 |  | P | P | P |
|  | TP01_0007 |  | P | P | P |
|  | *TP01_0008* |  | *Null* | *Null* | *Null* |
|  | TP01_0009 |  | ND | ND | ND |
|  | **…..** |  |  |  |  |
|  | TP01_1225 |  | P | P | P |
|  | TP01_1226 |  | P | P | P |
|  |  |  |  |  |  |
|  | **Locus** |  | **211T_A3 vs 211B_A3** | **951T_F44 vs 211B_A3** | **951T_F44 vs 211T_A3** |
| **Chromosome 2** | TP02_0003 |  | ND | ND | ND |
|  | TP02_0004 |  | ND | ND | ND |
|  | TP02_0005 |  | P | P | P |
|  | TP02_0006 |  | ND | ND | ND |
|  | TP02_0007 |  | P | P | P |
|  | TP02_0008 |  | ND | ND | ND |
|  | TP02_0010 |  | P | P | P |
|  | TP02_0011 |  | ND | ND | ND |
|  | **…..** |  |  |  |  |
|  | TP02_0953 |  | ND | ND | ND |
|  | TP02_0954 |  | -0.9 | P | 1.4 |
|  | TP02_0955 |  | P | P | P |
|  | TP02_0957 |  | P | P | P |
|  | TP02_0958 |  | P | P | P |
|  | TP02_0959 |  | P | P | P |
|  | TP02_0960 |  | P | P | P |
|  |  |  |  |  |  |
|  | **Locus** |  | **211T_A3 vs 211B_A3** | **951T_F44 vs 211B_A3** | **951T_F44 vs 211T_A3** |
| **Chromosome 3** | TP03_0001 |  | ND | ND | ND |
|  | TP03_0002 |  | P | P | P |
|  | TP03_0003 |  | P | P | P |
|  | TP03_0004 |  | ND | ND | ND |
|  | TP03_0005 |  | P | P | P |
|  | **…..** |  |  |  |  |
|  | TP03_0298 |  | P | P | P |
|  | **…..** |  |  |  |  |
|  | TP03_0867 |  | P | P | P |
|  | TP03_0868 |  | P | P | P |
|  | TP03_0869 |  | P | P | 2.0 |
|  | TP03_0870 |  | P | P | P |
|  | *TP03_0871* |  | *Null* | *Null* | *Null* |
|  | TP03_0872 |  | ND | ND | ND |
|  | *TP03_0873* |  | *Null* | *Null* | *Null* |
|  | TP03_0874 |  | ND | ND | ND |
|  | TP03_0875 |  | ND | ND | ND |
|  | *TP03_0877* |  | *Null* | *Null* | *Null* |
|  | TP03_0878 |  | ND | ND | ND |
|  | *TP03_0879* |  | *Null* | *Null* | *Null* |
|  | TP03_0880 |  | ND | ND | ND |
|  | TP03_0881 |  | P | P | P |
|  | TP03_0882 |  | P | P | P |
|  | TP03_0883 |  | P | 3.6 | P |
|  | TP03_0884 |  | P | P | P |
|  | TP03_0885 |  | ND | ND | ND |
|  | TP03_0886 |  | P | P | P |
|  | TP03_0887 |  | P | 5.7 | P |
|  | TP03_0888 |  | P | P | P |
|  | TP03_0889 |  | ND | ND | ND |
|  | TP03_0890 |  | -1.4 | 1.4 | 2.2 |
|  | TP03_0891 |  | P | P | P |
|  | TP03_0892 |  | P | P | P |
|  | TP03_0893 |  | P | P | P |
|  | *TP03_0930* |  | *Null* | *Null* | *Null* |
|  |  |  |  |  |  |
|  | **Locus** |  | **211T_A3 vs 211B_A3** | **951T_F44 vs 211B_A3** | **951T_F44 vs 211T_A3** |
| **Chromosome 4** | TP04_0001 |  | ND | ND | ND |
|  | TP04_0002 |  | P | P | P |
|  | TP04_0003 |  | P | P | P |
|  | TP04_0004 |  | ND | ND | ND |
|  | TP04_0005 |  | P | P | P |
|  | TP04_0006 |  | P | P | P |
|  | TP04_0007 |  | P | 1.2 | 1.7 |
|  | *TP04_0008* |  | *Null* | *Null* | *Null* |
|  | TP04_0923 |  | P | P | P |
|  | TP04_0009 |  | P | P | P |
|  | TP04_0010 |  | ND | ND | ND |
|  | TP04_0011 |  | ND | ND | ND |
|  | TP04_0013 |  | P | P | P |
|  | TP04_0014 |  | P | P | P |
|  | TP04_0015 |  | P | P | P |
|  | TP04_0016 |  | P | P | P |
|  | TP04_0018 |  | ND | ND | ND |
|  | TP04_0019 |  | ND | ND | ND |
|  | **…..** |  |  |  |  |
|  | TP04_0098 |  | P | P | P |
|  | TP04_0099 |  | P | P | P |
|  | **…..** |  |  |  |  |
|  | TP04_0406 |  | P | P | P |
|  | **…..** |  |  |  |  |
|  | TP04_0916 |  | P | P | P |
|  | TP04_0917 |  | P | P | P |
|  | TP04_0918 |  | P | P | P |
|  | TP04_0927 |  | P | P | P |
|  | TP04_0919 |  | P | P | P |
|  | TP04_0920 |  | P | P | P |
|  | TP04_0928 |  | P | P | P |
|  | TP04_0921 |  | P | P | 1.7 |
