# Supplementary material for: Expression Analysis of the Theileria parva Subtelomere-Encoded Variable Secreted Protein Gene Family
Source: PLoS One. 2009 Mar 27;4(3):e4839. doi: 10.1371/journal.pone.0004839 (PMC2657828; doi:10.1371/journal.pone.0004839)
Supplement: Table S2 — List of T. parva SVSP genes, indicating the presence or absence of a signal peptide and/or nuclear localisation signal (0.09 MB DOC) [file pone.0004839.s003.doc]

# Table S2

Predicted signal peptides and nuclear localisation signals in the SVSP family

| **SVSP** | **Signal peptide** | | **NLS** | | **SVSP** | **Signal peptide** | | **NLS** | |
| --- | --- | --- | --- | --- | --- | --- | --- | --- | --- |
|  | SignalP | SOSUI | NucPred | PSORTII |  | SignalP | SOSUI | NucPred | PSORTII |
| TP01_0004 | x | x | x | x | TP03_0881 | - | x | x | x |
| TP01_0005 | - | x | - | - | TP03_0882* | x | x | x | x |
| TP01_0006 | x | x | - | x | TP03_0883 | x | x | x | x |
| TP01_0007 | x | x | x | x | TP03_0884 | x | x | x | x |
| TP01_0008 | - | x | x | x | TP03_0885 | x | x | - | x |
| TP01_0009 | - | x | x | x | TP03_0886 | x | x | - | - |
| TP01_1225 | x | x | - | - | TP03_0887 | - | x | x | x |
| TP01_1226 | x | x | - | x | TP03_0888 | - | x | - | - |
|  |  |  |  |  | TP03_0889 | x | x | x | x |
| TP02_0003 | - | x | - | - | TP03_0890 | x | x | x | x |
| TP02_0004 | x | x | x | x | TP03_0891 | x | x | - | - |
| TP02_0005 | x | x | x | x | TP03_0892 | x | x | x | x |
| TP02_0006 | x | x | x | x | TP03_0893 | x | x | x | x |
| TP02_0007 | - | x | x | x | TP03_0930 | x | x | - | x |
| TP02_0008 | - | - | - | x |  |  |  |  |  |
| TP02_0010 | - | x | x | - | TP04_0001 | x | x | - | x |
| TP02_0011 | x | x | - | x | TP04_0002 | x | x | - | x |
| TP02_0953 | - | x | - | - | TP04_0003 | x | x | x | x |
| TP02_0954 | - | x | - | - | TP04_0004 | x | x | x | x |
| TP02_0955 | x | x | - | - | TP04_0005 | - | x | x | x |
| TP02_0957 | - | - | - | - | TP04_0006 | x | x | x | x |
| TP02_0958 | x | x | - | x | TP04_0007 | x | x | x | x |
| TP02_0959 | x | x | - | - | TP04_0008 | x | x | x | x |
| TP02_0960 | x | x | - | x | TP04_0009 | x | x | x | x |
|  |  |  |  |  | TP04_0010 | x | x | x | x |
| TP03_0001 | x | x | - | x | TP04_0011 | x | x | - | - |
| TP03_0002 | x | x | - | x | TP04_0013 | x | x | x | x |
| TP03_0003 | - | x | - | x | TP04_0014 | x | x | - | x |
| TP03_0004 | - | - | - | - | TP04_0015 | x | x | x | x |
| TP03_0005 | x | x | x | x | TP04_0016 | - | x | x | x |
| TP03_0298 | x | x | - | - | TP04_0018 | x | x | - | - |
| TP03_0867 | - | x | x | x | TP04_0019 | - | - | - | - |
| TP03_0868 | signal anchor | transmembrane region | - | x | TP04_0098 | x | x | x | - |
| TP03_0869 | x | x | - | x | TP04_0099 | x | x | - | x |
| TP03_0870 | - | x | x | x | TP04_0406 | x | x | - | x |
| TP03_0871 | x | x | x | x | TP04_0916 | x | x | - | - |
| TP03_0872 | x | x | - | x | TP04_0917 | x | x | x | x |
| TP03_0873 | x | x | x | x | TP04_0918 | x | x | x | x |
| TP03_0874 | x | x | x | x | TP04_0919 | x | x | - | - |
| TP03_0875 | x | x | x | x | TP04_0920 | x | x | - | x |
| TP03_0877 | - | - | - | x | TP04_0921 | - | x | - | - |
| TP03_0878 | x | x | x | x | TP04_0923 | x | x | x | x |
| TP03_0879 | x | x | x | x | TP04_0927 | x | x | x | x |
| TP03_0880 | x | x | x | x | TP04_0928 | x | x | x | x |
